# Health intersectoralism in the Sustainable Development Goal era: from theory to practice

**DOI:** 10.1186/s12992-020-0543-1

**Published:** 2020-02-20

**Authors:** Sameera Hussain, Dena Javadi, Jean Andrey, Abdul Ghaffar, Ronald Labonté

**Affiliations:** 1 Canadian Society for International Health, 1 Nicholas Street, Ottawa, Ontario K1N 7B7 Canada; 2 School of Epidemiology and Public Health, University of Ottawa, 600 Peter Morand Crescent, Ottawa, Ontario K1G 5Z3 Canada; 3 Alliance for Health Policy and Systems Research, World Health Organization, 20 avenue Appia, 1211 Geneva, Switzerland; 4 Faculty of Environment, University of Waterloo, 200 University Avenue West, Waterloo, Ontario N2L 3G1 Canada

**Keywords:** SDGs, Intersectoral action, Cross-sectoral engagement, Determinants of health, Multisectoral collaboration, Equity, Sustainable Development Goals, Intersectoral governance

## Abstract

In 2015, the United Nations’ (UN) Member States adopted a bold and holistic agenda of the Sustainable Development Goals (SDGs), integrating a vision of peace and prosperity for people and planet. Extensive work within, between, across sectors is required for this bold and holistic agenda to be implemented. It is in this context that this special article collection showcases multisectoral approaches to achieving SDG 3—Good Health and Well-Being—which, though focused explicitly on health, is connected to almost all other goals. A confluence of social and health inequities, within a context of widespread environmental degradation demands systems thinking and intersectoral action. Articles in this issue focus on the SDGs as a stimulus for renewed multisectoral action: processes, policies, and programs primarily outside the health sector, that have health implications through social, commercial, economic, environmental, and political determinants of health. Case studies offer critical lessons on effectively engaging other sectors to enhance their health outputs, identifying co-benefits and ‘win-wins’ that enhance human health.

## Background

The United Nations 2030 Agenda for Sustainable Development, with its 17 goals and 169 targets, is considered to be integrated and indivisible, a universal policy agenda that presents a plan of action for people, planet, and prosperity [1]. Few believe that the ambitions of this Agenda will be achieved in full, with the UN General Assembly in 2019 concerned that many of the targets are already off-track or lagging far behind [2]. Others dispute the indivisibility of the Agenda, noting how its economic growth assumptions are at perilous odds with its environmental imperatives [3, 4]. At the same time, in many countries the Agenda is galvanizing new efforts to implement all, or at least some, of its indisputably important goals. Nor is there any questioning that moving forward on the Agenda requires greater intersectoral action and multisectoral collaboration. As one example, SDG 3—Good Health and Well-being—is focused on ensuring healthy lives and promoting well-being through the life course and is contingent upon reducing inequities in social, commercial, cultural, economic, environmental, and political determinants of health [1, 5]. Despite broad acknowledgement of the foundational importance of intersectoral action, there remains a dearth of evidence on strategies and approaches for meaningful cross-sectoral engagement and implementation of cross-cutting policies specific to the SDGs [6, 7].

To begin to fill this lacuna, we issued a call for papers as part of a CIHR Health System Impact Fellowship and with support from the World Health Organization (WHO)‘s Alliance for Health Policy and Systems Research. The call focused on bridging the evidence to policy gap, emphasizing examples of efforts to improve health outcomes (SDG 3) through engagements with other goals and targets of the broader Agenda. The intent of this collection is to stimulate dialogue on setting priorities for intersectoral action, identifying co-benefits across sectors, and monitoring and managing collaboration. This introduction summarizes key points from the 12 articles included in the issue and discusses emergent themes [8–20].

## Main text

### Setting priorities

The article by Bennett et al. highlights how the SDG agenda challenges health policy-makers to identify a broader array of health policy and systems research priorities than those associated with the earlier Millennium Development Goals (MDGs). Drawing on interviews with policy makers, their study focuses on evidence gaps related to social protection, multisectoral collaboration, and new accountability measures. Policy-makers described the continued need for knowledge on strengthening primary health care and community-based health systems. With respect to multisectoral collaboration and the integration of further accountability measures, they wanted practical evidence on implementation processes, mechanisms, and expected outcomes. Engagement with non-traditional stakeholders outside of the health sector was largely regarded as unknown territory where further evidence could support decision-making and practice. Knowing the reaction of stakeholders within the health sector, especially health workers, to new accountability measures was also important to capture, with implementation research highlighted as a means of addressing these knowledge gaps.

Brolan et al. [17] further emphasize the importance of engaging participants from outside the health sector in both research and policy-making to address health-related goals. Their paper synthesizes findings from the Australian parliamentary inquiry of diverse stakeholders on SDG implementation impacts on Australia’s national and Overseas Development Assistance (ODA) SDG commitment (indicating a foreign and security policy orientation specific to the Indo-Pacific region). Improved understanding of, and reaction to the political determinants of health and SDG achievement may facilitate a paradigm shift in Australia’s development approach to the Pacific region, strengthening its ability to meet the development needs in the Pacific Region and its diverse sub-populations. But doing so requires genuine engagement with multiple sectors and applying an equity lens toward intersectoral policy implementation.

### Equity and the SDGs

Llop-Girones et al. [14] take the discussion on equity a step further by evaluating the capacity of national health information systems to monitor and measure health equity, using Mozambique as a case study. Focusing on the targets for SDG 3, the capacity for monitoring health inequalities was limited with significant gaps in the Health Information System, with notable exceptions around maternal, neonatal, and child health-related indicators. A paucity of information on disaggregated equity stratifiers prevents policy changes that could pave the way towards more comprehensive monitoring and measuring of health equity outcomes, with implications for overlaps with measures for other SDG targets. Lessons learned in Mozambique are likely transferable to other low- and middle-income countries (LMICs).

Delaney-Crowe et al. [12] examine whether health equity is considered in environmental policies and climate change mitigation efforts by reviewing documents on Australian environmental sector policies, in particularly around water – SDG 6, climate change – SDG 13, and marine ecosystems – SDG 14. While they find that health equity and health impacts are recognized as climate change effects, a more comprehensive approach to identifying health impact mitigation is needed. This, in turn, requires extensive and creative engagement across sectors, and greater policy coherence between jurisdictions. National coordination will be needed to protect existing parks and water and sanitation systems from future threats.

Ramirez-Rubio and colleagues [9] offer an approach to facilitate policy coherence and coordination by designing an operational framework that bridges urban policies (SDG 11) and public health planning (SDG 3). By using Health in All Policies in the context of SDG implementation, they explore different approaches to better anticipate the impacts of urban design on health and health equity. These approaches include capturing the quantitative burden of disease at city-levels, undertaking health impact assessments, and involving citizen and other stakeholders to inform an integration of health recommendations within urban policy. Their framework clarifies links between the social determinants of health, environmental exposures, behaviour, health outcomes, and urban policies corresponding to 15 SDGs and 38 targets. Their work supports design of policies that promote active transportation, urban greening, and healthy public open spaces, drawing on examples from several countries.

### Implementation of intersectoral action

Having frameworks to guide intersectoral action is essential, but implementation can remain a challenge despite knowing downstream health impacts of investment in non-health sectors. McGuire et al. [10] take on this challenge by reviewing co-financing models for intersectoral action, usually involving health, education, and social care sectors. Cost-effectiveness estimates are complicated when impacts do not fall strictly within one sector’s purview, and the co-benefits can then be more difficult to sell. Innovative models from six countries suggest that pooling budgets across sectors, including non-public and international payers, can offset flatlining global development assistance for health, and optimize public spending.

Another financing mechanism explored in the special issue is taxation. Hangoma and Surgey [18] examine sin taxes in Zambia, and the importance of intersectoral action to identify its co-benefits. Such taxes undertaken to reach health targets may nonetheless be challenged by arguments of their negative impact on other SDG targets related to employment, economic growth, and poverty elimination. Responding to this potential challenge, their paper examines the possible effect of Zambia’s sugar tax intended to reduce non-communicable diseases (NCDs) (SDG 3.4), in light of commitments to increase employment (SDG 8.5) and economic growth (SDG 8.1). They found no reliable evidence of contradiction between these targets, at least at this time, likely owing to the low level of sugar taxation. They caution about the need for care in setting taxation policies in order to maximize co-benefits, arguing that this may demand moderation in tax measures.

Wright and colleagues [15] further expand on contradictions and conflicts of interest in intersectoral action by examining the circular economy (CE) and its potential positive and negative health impact. CE counters the traditional model of “take, make and dispose” and calls for cyclical use of materials. Implementation of CE in LMICs is often driven by poverty and unemployment and can impose health risks including exposure to hazardous and toxic work environments, conditions, emissions, and materials, as well as infectious diseases. Despite these challenges, CE has the potential to contribute toward SDG achievement, especially by mitigating climate change and reducing poverty in LMICs, insofar that it is contextualized and implemented appropriately. That policymakers, industry, and health sectors must define the mechanisms to protect vulnerable populations from potential negative health impacts is an imperative is clear—and such decisions must be made with full agency from the public to move toward responsible consumption and production (SDG 12) and positive synergies for other SDGs.

Echoing some of the critiques of Agenda 2030’s contradictory reliance on continued economic growth to achieve many of the goals, Meurs et al. [16] question whether LICs can attain the minimal suggested level of health spending per capita required to achieve SDG 3 by fulfilling SDG 8.1’s 7% annual economic growth target. They focus on three African countries (Malawi, Uganda, and Tanzania) and show starkly that to fill the health spending funding gap would require unprecedented, unrealistic, and environmentally challenging levels of economic growth. Moreover, health spending from domestic revenue generation would need to be given much greater priority in governments’ budgetary allocations. They also find that recent IMF programs and policy advice to the three countries still emphasize fiscal consolidation and regressive taxation measures, affecting health spending directly and key health determinants indirectly. Their paper underscores the importance of global financing (including all HICs meeting their 0.7% target of ODA spending) if SDG 3 and other social and environmentally protective SDGs are to come near their fulfilment.

### Meso and micro level stakeholder engagement

Although global in reach and national in obligation, SDGs are experienced locally. This implies a necessity to negotiate co-benefits and mitigate threats with stakeholders across multiple governance levels. Thus far, papers in this special issue have addressed primarily macro-level (national or internationally comparative) approaches. Tan et al. [11] explore micro- level localisation on intersectoral action to support the SDGs through seven urban-based case studies in Malaysia, two of which are described in some detail. Their research focuses on the use of systems thinking and the creation of causal loop diagrams to create ‘placially explicit systems-based’ SDG activities and measures to overcome difficulties in basing actions solely on generalized (global- or national-level) strategies, targets, and indicators. They suggest that the tools field-tested in their case studies provide new and transdisciplinary ways to understand and act upon the linkages inherent in SDGs and their targets, across sectors and at multiple scales, but with specific reference to improving urban health and well-being.

Garcia and colleagues [13] offer a meso-level solution by demonstrating the role of community courts (rather than criminal courts) in tackling multiple SDG targets in Canada. Although focusing principally on SDG 3.5, their detailed analysis of a community court in Vancouver’s drug-troubled downtown area touches on how the court’s ‘problem-solving’ work involves tight connections between health, justice, and social service systems—and can address case by case needs and circumstances leading individuals to criminal behaviour. In doing so, the court not only diverts cases away from the criminal system; it is also able to take into account other SDGs that bear on Vancouver’s increasing number of drug-related infractions and opioid overdoses, including targets associated with SDGs 1, 5, 10, 16, and 17. Optimistically, the authors conclude that community courts could provide a pathway to address Canada’s (and other countries’) drug-related public health emergencies by focusing on the underlying conditions leading to problematic drug use and related crimes.

The momentum established by the concepts discussed across these papers – the Sustainable Development Agenda, Health in All Policies, Planetary Health, the circular economy, systems thinking, and health equity impact assessments – has created a unique opportunity for action. National, regional, and international policy-making bodies are pushing for equity-driven, multisectoral, and transparent policies that promote health beyond the health sector. Hussain et al. provide in this issue an analysis of Voluntary National Reviews of the SDGs juxtaposed with the priorities of marginalized communities in nine countries. With a specific focus on new mechanisms for SDG operationalization, they explore the political will and likelihood of success in Leaving No One Behind. The collection’s concluding commentary by Al-Mandhari et al. demonstrates [8] this shift by highlighting the WHO’s Eastern Mediterranean Regional Office’s (EMRO) plans to accelerate progress towards the SDGs. The adoption of a whole-of-government approach, partnerships with civil society, and investing in leadership to support intersectoral action are all part of EMRO’s revitalized efforts.

## Conclusion

This special article collection on “Health in the SDGs: Intersectoral Action for Health” fits with Globalization and Health’s aims of exploring how “globalization processes affect health through their impacts on health systems and the social, economic, commercial, and political determinants of health.” The mechanisms and pathways through which health is affected as a result of globalization can be leveraged through intersectoral action to enhance rather than inhibit health. Evidence is lacking on concrete approaches for successful intersectoral action; the papers in this special issue are part of an effort to expand the knowledge base and to stimulate discussion on methodologies to address this gap, identify stakeholders that need to be engaged, and improve the abilities to work across macro, meso, and micro levels of policy and programmatic action – all of which need to, and can be, tested across different settings.

The SDGs have created a unique opportunity for sectors to seek support from one another and identify co-benefits while managing contradictions and threats. Policy-makers and practitioners are grappling with these realities in their day-to-day work. Research that cuts across disciplinary boundaries and is embedded within the implementation of cross-cutting policies is critical in ensuring that efforts to achieve SDG targets in one sector are optimized to support achievement of targets in another – recognizing that the inherent contradictions within some of the SDGs (notably those related to economic growth and environmental sustainability) will need to be acknowledged and addressed. In the instance of climate change, for example, the challenge is not ‘balancing’ economic and environmental dimensions since, as the contribution by Meurs et al. cautions, this risks default precedence being given to growth when, 4 years after their promulgation, it is the environmental SDGs that are amongst the most off-track [16].

The challenges now are even greater than in 2015, when the Agenda itself acknowledged the need to confront the “enormous disparities of opportunity, wealth and power in the world” [1], a world that has since seen a rise in xenophobia and authoritarianism. As some governments move progressively forward with the Agenda, growing numbers of civil society organizations have emerged, many with young people and women in leadership roles, challenging nations to deliver on their promises. Multilateral collaboration requires increasing attention to, and spaces for, civil society engagement, lest the SDGs falter on an adherence to the interests of powerful private economic actors [20]. We have an ever-growing knowledge base on the impact of the different determinants of health; we continue to improve our understanding of how to apply this knowledge to drive intersectoral action and ensure the consideration of health in all policies; and we must now respond more effectively to social movement demands for equity in our social and economic development, and sustainability in our environmental resources. The hopeful promise of the SDGs remains as vital today as it did in 2015, with the next 10 years crucial for implementation.

## Data Availability

Not applicable.
